# Fabrication of Composite Hydrogels Based on Soy Protein Isolate and their Controlled Globular Protein Delivery

**DOI:** 10.1002/gch2.201900030

**Published:** 2019-05-27

**Authors:** Naipu He, Xiunan Chen, Li Wang, Jing Wen, Yuhong Li, Qi Cao, Zaiman Liu, Baiyu Li

**Affiliations:** ^1^ School of Chemical and Biological Engineering Lanzhou Jiaotong University Lanzhou 730070 China

**Keywords:** composite hydrogels, controlled release, polyacrylic acid, protein delivery, soy protein isolate

## Abstract

Soy protein isolate (SPI) protein/polymer composite hydrogels (PPCGs) are fabricated in a urea solution of SPI using acrylic acid as monomer, ammonium persulphate (APS) as initiator, and *N*,*N*‐methylenebisacrylamide (BIS) and glutaraldehyde (GA) as cross‐linking agents. The scanning electron microscope (SEM) results show that SPI/polyacrylic (PAA) composite hydrogels formed network structure. In particular, in the absence of cross‐linking agent (GA), the network structure of composite hydrogels is also formed by BIS cross‐linking chains of PAA and the hydrophobic interactions between peptides from SPI and chain of PAA. In addition, composite hydrogels have good water absorption and present excellent pH sensitivity. Composite hydrogels adsorb bovine serum albumin (BSA) with higher adsorption capacity. BSA is the control released in pH 7.4 buffers and the accumulative release ratio achieved is 90%. It will be expected that these protein/polymer composite hydrogels could be applied for drug sustained release materials.

## 1 Introduction

Hydrogels formed through cross‐linking of hydrophilic polymer chains, the network of 3D swelling in aqueous solution, and holding large volumes of water, but undissolved in water.^1^, ^2^, ^3^ Because of the water‐rich nature of hydrogels, they have potential application in controlled biomacromolecules delivery. In addition, hydrogels present an aqueous environment that prevents protein denaturation.^4^, ^5^, ^6^

Biopolymer‐based hydrogels have recently attracted interest in drug delivery applications. Biopolymers were used to prepare hydrogels as drug carriers due to the advantages of renewability, biodegrade ability, low toxicity, and biocompatibility.^7^, ^8^, ^9^ The typical naturally occurring biopolymers are polysaccharides such as chitosan, cellulose, and alginate. Biopolymer‐based hydrogels possess a high content of functional groups including hydroxyl, amino, and carboxylic acid groups. These functional groups are benefited to form the networks of hydrogels, and they further bioconjugate with cell targeting agents.^10^

Proteins are abundant sources of biodegradable and biocompatible natural polymers. They were considered as potential candidate to fabricate environment‐friendly polymeric materials.^11^, ^12^, ^13^ Proteins were also used to prepare gel by self‐assembly or aggregation.^14^, ^15^, ^16^ In addition, proteins can also be employed as building blocks in the design of composite hydrogels consisting of synthetic polymeric and peptide structural elements. Hydrogel based on protein were applied in biomedicine field as tissue engineering materials and drug delivery, because they are easily degraded by the body and display a high biocompatibility.^17^, ^18^, ^19^

Soy protein isolate (SPI), a protein with reproducible resource, good biocompatibility, and biodegrade ability, has a significant potential in the bioscience and biotechnology.^20^, ^21^, ^22^ A large number of biocompatible protein composite materials based on SPI was constructed by chemical grafting, blending, and self‐assembly.^23^, ^24^, ^25^ In general, native SPI was dissolved in alkaline solution in which native SPI was denatured and unfolded to form peptide chains. Unfolded protein formed peptide chains which improved the solubility of protein in water. Peptides were employed as excellent building block to fabricate hydrogel.^26^, ^27^, ^28^ In addition, incorporation of peptides into hydrogel presents a number of functions including cell‐binding, growth factor binding, surface binding, electroactivity, and so on.^29^ For instance, hydrogel prepared from SPI showed a good electroactivity.^30^

Soy protein hydrogels exhibited the better mechanical properties after the introduction of natural polysaccharide derivative as a macromolecular cross‐linking agent.^31^ The gel polymer electrolyte based on the SPI/poly(vinyl alcohol) composite nanofiber membranes presented high ionic conductivity, excellent compatibility with lithium electrode, and good electrochemical stability.^31^ Hydrogel based on SPI has good mechanical strength and was found to have a potential biomedical application for site‐specific drug delivery.^33^, ^34^ Hydrogel based on SPI was also investigated as 3D cell‐laden bio‐printing materials.^35^, ^36^ All the reports reveal that gelation of SPI improved the properties of SPI and greatly broadened the application, especially as a biocompatibile materials.

As a water‐soluble polymer, polyacrylic acid (PAA) was commonly used as building block to prepare hydrogel. PAA used to enhance the properties of hydrogels and applied in releasing of drugs self‐healing property and oral insulin delivery.^37^, ^38^, ^39^, ^40^, ^41^

In the current paper, since they are also abundant in nature, renewable, nontoxic, and relatively cheap, soy protein isolate (was employed as raw materials to fabricate a new kind of SPI/PAA composite hydrogels. The structure of hydrogels was observed by scanning electron microscope (SEM). Furthermore, the swelling rate of composite hydrogels in water and the release behavior of globular protein bovine serum albumin (BSA) from the hydrogels were investigated in details. Prior to preparation of SPI/PAA composite hydrogels, SPI was denatured and unfolded in the urea solutions to form peptide chains. SPI/ PAA composite hydrogels (PPCGs) were obtained in the urea solution by using acrylic acid (AA) as the monomer, ammonium persulphate (APS) as the initiator, *N*,*N′*‐methylene bisacrylamide (BIS) as the cross‐linking agent in presence of or in absence of the other cross‐linking agent glutaraldehyde(GA). As cross‐linking agent, *N*,*N′*‐methylene bisacrylamide can participate in the polymerization of monomers to form the network of synthetic polymers. In addition, in presence of the other cross‐linking agent glutaraldehyde, GA linked peptides from SPI with synthetic polymers. In absence of GA, peptides from SPI self‐assembled with synthetic polymers to form the network structure. The results revealed that SPI/PAA hydrogels exhibit good swelling behavior in the water and excellent controlled release for globular protein.

## 2 Results and Discussion

### 2.1 Preparation and Characterization of SPI/PAA Composite Hydrogels

SPI/PAA composite hydrogels were prepared in the urea solution. SPI powder was prior dissolved in the urea solution to form unfold peptides as a stock protein solution. The monomer acrylic acid and the cross‐linking agent *N*,*N′*‐methylene bisacrylamide were initiated by ammonium persulphate to form the prenetwork structure in the stock protein solution. At the same time, in presence of the other cross‐linking agent glutaraldehyde (GA), GA linked peptides from SPI with synthetic polymers to form composite network structure. Besides, in absence of GA, the network structure of SPI/PAA composite hydrogels was fabricated by self‐assembly of peptides from SPI with synthetic polymers.

As shown in **Table** **1**, SPI/PAA composite hydrogels were prepared at the difference ratio of SPI to monomer AA, including SPI:AA = 1:4, 1:6, 1:8, 1:10, and 1:12, respectively. The high critical ratio of SPI to AA was demonstrated as 1:4 to form composite hydrogels. At the lower concentration of monomer AA, hydrogel cannot be formed and the floccule can be formed. Meanwhile, at the ratio of SPI:AA as 1:4 and 1:6, composite hydrogels were formed, but they exhibited the lower tenacity and became pieces easily. With increasing the concentration of AA, the hydrogels became harder and the morphology revealed tighter.

**Table 1:** Preparation of protein/polymer composite hydrogels

Sample ^a)^	AA [mL]	SPI:AA [g g^−1^]	BIS [g]	GA [mL]	Gelation
PPCGs1	1.0	1:4	0.05	0.1	Hydrogel
PPCGs2	1.5	1: 6	0.05	0.1	Hydrogel
PPCGs3	2.0	1:8	0.05	0.1	Hydrogel
PPCGs4	2.0	1:8	0.01	0.0	Hydrogel
PPCGs5	2.0	1:8	0.01	0.1	Hydrogel
PPCGs6	2.0	1:8	0.05	0.0	Hydrogel
PPCGs7	2.5	1:10	0.05	0.1	Hydrogel
PPCGs8	3.0	1:12	0.05	0.1	Hydrogel

^a)^ A stock SPI solution was prepared in 8 mol L^−1^ of urea solution by dissolving 0.25 g of SPI powder in 20 mL of urea solution at room temperature. The weight of APS was kept as 0.05 g. PPCGs were performed in the stock SPI solution at 60 °C.

**Figure 1:**
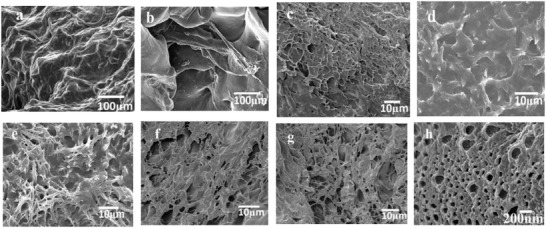
The SEM of SPI/PAA composite hydrogels (PPCGs). a) PPCGs1, b) PPCGs2, c) PPCGs3, d) PPCGs7, e) PPCGs8, f) PPCHs4, g) PPCGs5 and h) PPCGs6.

**Figure 2:**
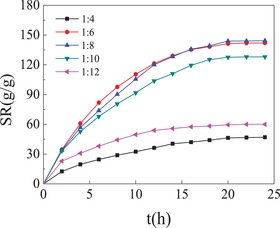
The ratio of SPI to monomer AA effecting on the swelling ratio of SPI/PAA composite hydrogels. SPI/PAA composite hydrogels were prepared in presence of cross‐linking agent BIS (0.05 g) and GA (0.1 mL) at the varied ratio of SPI to monomer AA. PPCGs1, SPI: AA = 1:4; PPCGs2, SPI: AA = 1:6; PPCGs3, SPI: AA = 1:8; PPCGs7, SPI: AA = 1:10; PPCGs8, SPI: AA = 1:12. The weight of dried hydrogels was determined as 0.1 g in the all experiments.

**Figure** **1** showed the SEM photographs of SPI/PAA hydrogels. Keeping the concentration of cross‐linking agents BIS and GA as invariable, the morphologies of PPCGs were influenced by the difference ratio of SPI to monomer AA (Figure 1a–c,g,h). With increasing the concentration of AA, more networks were observed at the surface of hydrogels. At the lower concentration of monomer AA, the networks were not formed (**Figure** **2**ab). Figure 1a showed that some nanorods were formed due to self‐assembly of the unfold peptides from SPI. At the higher concentration of monomers, the networks were observed (Figure 1b,c,g,). At the ratio of SPI to AA as 1:8, the well‐defined network structures were observed under SEM (Figure 1h). At the ratio of SPI to AA as 1:8, the well‐defined network structures were observed under SEM (Figure 1h).

When the GA was used as cross‐linking agent to cross‐link SPI, –CHO, and –NH_2_ would undergo aldol condensation reaction to produce Schiff base. Peptides from SPI covalently linked with the networks of synthetic polymers to form hybrid hydrogels. The results revealed that hybrid hydrogels with networks structure and uniform cavities were prepared in presence of or in absence of the second cross‐linking agent (GA)(Figure 1h). In the absence of the second cross‐linking GA, SPI/PAA composite hydrogels could also be obtained (Figure 1c,h).

In the present paper, SPI was completely dissolved in the urea solutions which are alkaline conditions. In alkaline conditions away from the isoelectric region, native SPI was denatured and unfolded, and became peptide chains. Sulfhydryl and hydrophobic groups on the chain of peptides will be exposed in the solution. In the present paper, composite SPI/PAA hydrogels were prepared in the alkaline conditions. It was hypothesized that the unfolded peptide chain associated with chains of PAA by noncovalent interactions such as hydrophobic interactions to form peptide/PAA hybrid networks. Therefore, in absence of the second cross‐linking agent glutaraldehyde, since the unique self‐assembly characteristic of peptide, peptides from SPI self‐assembled with the networks of synthetic polymers by hydrophobic interactions to form hybrid networks.

### 2.2 The Swelling Properties of SPI/PAA Composite Hydrogels

SPI/PAA composite hydrogels were prepared at the difference ratio of SPI to monomer AA, including SPI:AA = 1:4, 1:6, 1:8, 1:10, and 1:12, respectively. The cross‐linking agents BIS and GA were presented as 0.05 g and 0.1 mL, respectively. As shown in Figure 2, PPCGs prepared by the difference ratio of SPI to monomer AA exhibited the difference swelling behavior. PPCGs2 (SPI:AA = 1:6) and PPCGs2 (SPI:AA = 1:8) exhibited the excellent swelling ratio (140%) and achieve the swelling equilibrium within 20 hours. The swelling ratio of PPCGs1 (SPI:AA = 1:4) was unsatisfactory. The AA content was less and therefore did not support 3D network construction. With the increase in the content of AA, and the increase in the swelling ratio of hydrogels, the swelling rate reached the maximum, i.e., 140 g g^−1^. Nevertheless, as the weight ratio of SPI to monomer AA reached 1:12, the ratio of PPCGs8 decreased to 60 g g^−1^. The AA content was so much leading to the porous not only intensive but also small, so the water was difficult to enter into inside of hydrogels.

**Figure 3:**
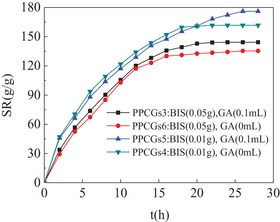
The cross‐linking agents effecting on the swelling ratio of SPI/PAA composite hydrogels. SPI/PAA composite hydrogels were prepared at the ratio of SPI to monomer AA as 1:8 in presence of cross‐linking BIS and GA. PPCGs3, BIS: 0.05 g and GA: 0.1 mL; PPCGs4, BIS: 0.01 g and in absence of GA; PPCGs5, BIS: 0.01 g and GA: 0.1 mL; PPCGs6, BIS: 0.05 g and in absence of GA. The weight of dried hydrogels was determined as 0.1 g in the all experiments.

As shown in **Figure** **3**, the cross‐linking agents effecting on the swelling ratio of SPI/PAA composite hydrogels were investigated. In the present work, network structures of PPCGs were formed by cross‐linking using BIS and GA. And it was amazing that network structure of PPCGs was also obtained in absence of the second cross‐linking agent GA. When the concentration of BIS was 0.1 g in presence of or in absence of the second cross‐linking GA, the hydrogels exhibited higher swelling ratio. In addition, in absence of the second cross‐linking agent GA, the swelling ratio and equilibrium time of hydrogels was similar to that of hydrogels in presence of GA. It could be concluded that the abundance of cross‐linking agents led to the increase of the cross‐linking points in hydrogels. Therefore, the overstocked cross‐linking density influenced the swelling ratio. However, if the concentration of cross‐linking agent was insufficient, the hydrogels could not form the effective cross‐linking networks.

**Figure 4:**
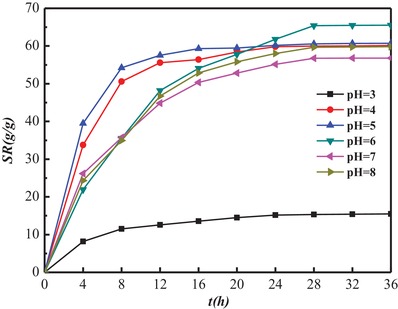
The swelling ratio of hydrogels in the buffer solutions varied pH from 4 to 8. The dried PPCGs8 was selected and determined as 0.1 g in the performance.

The swelling ratio of hydrogels in the buffer solutions varied pH from 4 to 8 was investigated (**Figure** **4**). In the solution pH = 3, the swelling ratio was very low. In the solution pH > 3, the hydrogels have the good swelling behavior. The hydrogels were composed of PAA and peptides from SPI. Since PAA and peptides are conventional and typical polyelectrolyte, chains stretch of PAA and peptides were influenced by pH in solution. Similarly, the network structure of hydrogel was also changed by pH, which would result to the swelling behavior of hydrogel in the solution varied by pH.

SPI are polyampholytic polymers, and the variety of pH values will influence the association and dissociation behaviors of protein in aqueous solution. At a pH value below their Isoelectric point (pI), protein exhibit a positive net charge, while at a pH above their pI, protein bear a negative net charge. pI of the native SPI is considered as pH 4.5. SPI was coagulated rather than dispersed in water at the pH near its isoelectric point (pI4.5). It was reported that the swelling ratio of the individual protein hydrogels reaches its minimum in a swelling medium at a pH close to the isoelectric point of the native proteins.^42^ Furthermore, in extreme acidic and alkaline conditions, the strong repulsive forces of highly negative or positive charges were present along protein chains, which prevented protein molecules from associating.^43^

In the present paper, in the solution pH = 3, the swelling ratio of the hydrogels reaches its minimum. In this condition, pH value below pI (pH 4.5) of SPI, peptide chains of SPI bear a positive net charge which prevented peptide chains from associating. In addition, the side groups (–COOH) of PAA cannot completely ionize and form the strong hydrogen bonds between PAA chains under the conditions, which leads to decrease in hydrophilicity of PAA chains. Because of the charged property of peptide chains of SPI and the ionized degree of side group (–COOH) of PAA decreasing, it makes sense that the swelling ratio of SPI/PAA composite hydrogels reached its minimum at acidic conditions (pH = 3).

### 2.3 The Loading Behavior of Hydrogels (PPCGs) for Globular Protein

Bovine serum albumin was employed as model protein to investigate load capacity of hydrogels for globular protein. All of performances were carried out in 0.01 mol L^−1^ phosphate buffer solution at pH = 7.4(PBS7.4). Protein concentration effecting on the load capacity of hydrogels was explored in PBS7.4 with different protein initial concentration, i.e., 0.5, 1, 3, 5 mg mL^−1^, respectively.

**Figure 5:**
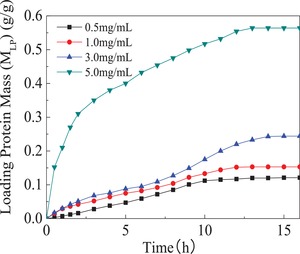
SPI/PAA composite hydrogels loading protein in PBS7.4 with different protein initial concentration. 0.1 g of dried PPCGs3 was selected to load protein. The initial concentration of protein was prior determined as 0.5, 1, 3, 5 mg mL^−1^, respectively. All performances were carried out in 0.01 mol L^−1^ phosphate buffer solution at pH = 7.4(PBS7.4).

As shown in **Figure** **5**, the loaded protein mass (*M*_LP_) of hydrogels was saturated within 13 hours. Additionally, with the increase in protein initial concentration, the loading weight of protein increases. In the protein solution with higher BSA initial concentration, hydrogels exhibited the higher loading capacity for protein. The loaded protein mass was the biggest in the 5 mg mL^−1^ BSA solution and calculated as 0.56 g g^−1^. The protein‐loaded hydrogels with different loaded BSA mass (M_LP_) were prepared in PBS7.4, and M_LP_ was determined as 0.12, 0.15, 0.24, and 0.56 g g^−1^, respectively. The results showed that the composite hydrogel have good loading profile for protein. The porosity and pore size of the composite hydrogel helped to load macromolecules protein.

In addition, introduction of peptides from SPI in the structure of composite hydrogel enhanced the affinity of hydrogel for protein, which increased the loading capacity of hydrogel for protein. Therefore, we could get conclusion that SPI/PAA composite hydrogels have potential advantages as loading materials for globular proteins.

### 2.4 Release of Protein from Hydrogels (PPCGs)

**Figure 6:**
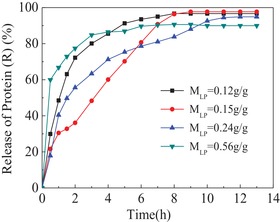
Release of protein from protein‐loaded hydrogels with different loaded protein mass (MLP) in PBS7.4. All performances were carried out in 0.01 mol L^−1^ phosphate buffer solution at pH = 7.4(PBS7.4).

**Figure** **6** showed that the release of the model protein BSA from the composite hydrogels occurred spontaneously in PBS 7.4. The release speed of the protein from protein‐loaded hydrogels with higher loaded protein mass was fastest (*M*_LP_ = 0.56 g g^−1^) compared to other hydrogels. BSA was released rapidly from protein‐loaded hydrogels in initial 4 hours. Afterward, the release speed was slow and reached equilibrium within 8 hours. The cumulative drug release data suggest that BSA is almost completely released from the hydrogel (nearly 100%), and sustained release formulations are much excellent.

This phenomenon contributed to prolong the medicine remedy time and promoted drug assimilate further, reducing the times of dose and enhancing the use efficiency of medicines. The porosity and pore size of the composite hydrogel have become significant channels to the diffusion of protein. All results indicated that controlled release of proteins from these composite hydrogels may be suitable drug delivery for biomacromolecules.

The hydrogel enables the efficient encapsulation of the protein cargo, and enables protein release at a controlled release rate in aqueous phase in which denaturation of proteins was prevented.^44^ Other biopolymer‐based hydrogels have been also explored for controlled release of proteins. For example, chitosan‐based hydrogels were prepared, allowing the safe incorporation of BSA and enabling the sustained release of protein.^45^, ^46^ Alginate composite hydrogels also showed great potential for the sustained release of protein.^47^, ^48^ Alginate/chitosan hydrogel microcapsules were modified with methoxy poly(ethylene glycol) (MPEG) to improve protein repellency and biocompatibility.^49^ Biopolymer‐based hydrogels, including soy protein isolate, are intrinsically biodegradable and nontoxic. These unique properties offer great potential for the utilization of hydrogels in applications for protein delivery.

## 3 Conclusions

In this present work, a new class of composite hydrogels based on soy protein isolate was successfully fabricated with varying concentration of synthetic polymer polyacrylic acid to develop a controlled delivery system for globular protein. Prior to preparation of SPI/PAA composite hydrogels, SPI was denatured and unfolded in the urea solutions to form peptide chains. The networks structure of hydrogels was formed using *N*,*N′*‐methylene bisacrylamide as the cross‐linking agent, and in presence or absence of the second cross‐linking agent glutaraldehyde (GA). In the absence of the second cross‐linking GA, SPI/PAA composite hydrogels were fabricated by BIS cross linking chains of PAA and the hydrophobic interactions between peptides from SPI and chain of PAA. The high critical ratio of SPI to AA was demonstrated as 1:4 to form composite hydrogels. With the increase of the ratio of SPI to monomer AA, the well‐defined network structures were observed under the scanning electron microscope. All hydrogels exhibited the excellent swelling capacity in water, and the maximum of swelling rate was calculated as 140 g g^−1^. Furthermore, the hydrogels loaded model globular protein BSA in and controlled macromolecules delivery phosphate buffer solution at pH = 7.4(PBS7.4). BSA was almost completely released from the hydrogel (nearly 100%) and reached equilibrium within 8 hours. The composite hydrogels based on soy protein isolate will be expected as a suitable drug delivery for globular proteins.

## 4 Experimental Section

*Materials*: Soy protein isolate was purchased from Changchun Jinyuan Industrial, China. Acrylic Acid was purchased from Chinese Medicine Company. *N*, *N*‐methylene bisacrylamide and bovine serum albumin were purchase from Shanghai Sangon Biotechnology Co. Ltd., China. Ammonium persulphate was purchased from Yantai Chemical Co. Ltd., China. Glutaraldehyde was purchased from Shanghai Zhongqin Chemical Reagent Co. Ltd., China. Other reagents were analytical grade and used without further purification.

*Fabrication of SPI/PAA Composite Hydrogels*: A stock SPI solution was prepared in 8 mol L^−1^ of urea solution by dissolving 0.25 g of SPI powder in 20 mL of urea solution at the room temperature. BIS and APS were prior dissolved in 1 mL of the distilled water, respectively. SPI/PAA composite hydrogels were prepared as follows: monomer AA, a solution of BIS, a solution of APS and GA were successively added into the stock SPI solution. The mixture solution was constantly stirred and heated at 60 °C. SPI/PAA hydrogels were formed after reacting for 2 h. The resulting SPI/PAA hydrogels were moved into distilled water to remove unreacted monomer and cross‐linking agent for a week, and the distilled water was renewed for 24 h. The hydrogels were moved into ethyl alcohol for 48 h and ethyl alcohol was renewed per 4 h. A fraction of the obtained hydrogel was dried at 50 °C in vacuum. The dried hydrogels were prepared due to their high swelling capacity and used for the determination of the swelling rate.

*Scanning Electron Microscope*: SEM (JSM‐6701F from Hitachi JEOL Co. Ltd., Tokyo, Japan) was used to observe the surface topography of dried hydrogels. Samples of dried hydrogels were prepared by freeze‐drying and then on the copper cylinder to metal spraying samples surface for 20 min. The surface of dried hydrogels was observed by SEM under 5 kV.

*Determination of the Swelling Rate (SR) of SPI/PAA Composite Hydrogels in Water*: The dried hydrogels were put into distilled water to swell. The swelling hydrogels were removed from water at predetermined time intervals to measure the weight of hydrogels. Then hydrogels were moved into the distilled water again and continued to swell. Swelling equilibrium was reached when the weight of the hydrogels were kept constant. The swelling rate of all samples was calculated as the follows

$$\mathrm{SR}\left(\mathrm{g/g}\right)\;=\;\left(W_{t}-W_{0}\right)/W_{0}$$

The *W*_0_ was the weight of dried hydrogels and *W*_t_ was the weight of swelling hydrogels.

*SPI/PAA Composite Hydrogels Loading Globular Protein*: Bovine serum albumin was employed as model globular protein to investigate the loading capacity of hydrogels for globular protein. Protein solutions were prior prepared in 0.01 mol L^−1^ phosphate buffer solution at pH = 7.4(PBS7.4). The initial concentration of protein was determined as 0.5, 1, 3, 5 mg mL^−1^, respectively. The protein‐loaded hydrogels were prepared as follows. First, 0.1 g of dried hydrogels was completely swelled in PBS 7.4. Subsequently, the swelling hydrogel was moved into 50 mL solution of BSA. During immersion, 2 mL samples were removed from solution of BSA at predetermined time intervals for analysis. The absorbance of the samples was determined at 278 nm by ultraviolet spectrophotometer (UV2102P) from which the concentration of protein in the PBS7.4 was determined. The loaded protein mass (*M*_LP_) was calculated as the following formula

$$M_{\mathrm{LP}}^{}\;\;=\;\;(Co\;\;-\;\;C)\;\;\times \;\;V/W_{0}$$

The C_0_(mg mL^−1^) was the initial concentration of protein solution and *C* was the instantaneous concentration of protein solution. The *V* was the volume of protein solutions and *W*_0_ was the weight of dried hydrogels.

*SPI/PAA Composite Hydrogels Controlled Release for Globular Protein*: The BSA‐loaded hydrogels were put into PBS 7.4 to investigate the hydrogels release behavior for globular protein. During immersion, 2 mL samples were removed from PBS 7.4 at predetermined time intervals for analysis. The absorbance of the samples was determined at 278 nm by ultraviolet spectrophotometer (UV2102P) from which the concentration of protein released from hydrogels was determined. The cumulative release ratio of protein (R (%)) in PBS 7.4 was calculated as:

$$R(\%)\;\;=\;\;M{\mathrm{/M}}_{\mathrm{LP}}^{}\;\;\times \;\;100\%$$

where *m* was the weight of BSA which released from hydrogels and *M*_LP_ was the loaded protein mass.

## Conflict of Interest

The authors declare no conflict of interest.
